# Circulating miR-184 is a potential predictive biomarker of cardiac damage in Anderson–Fabry disease

**DOI:** 10.1038/s41419-021-04438-5

**Published:** 2021-12-11

**Authors:** Irene Salamon, Elena Biagini, Paolo Kunderfranco, Roberta Roncarati, Manuela Ferracin, Nevio Taglieri, Elena Nardi, Noemi Laprovitera, Luciana Tomasi, Marisa Santostefano, Raffaello Ditaranto, Giovanni Vitale, Elena Cavarretta, Antonio Pisani, Eleonora Riccio, Valeria Aiello, Irene Capelli, Gaetano La Manna, Nazzareno Galiè, Letizia Spinelli, Gianluigi Condorelli

**Affiliations:** 1 Humanitas Research Hospital – IRCCS, 20089 Rozzano, (MI) Italy; 2 Department of Biomedical Sciences, Humanitas University, 20090 Pieve Emanuele, (MI) Italy; 3 Cardiology Unit, St. Orsola Hospital, IRCCS Azienda Ospedaliero-Universitaria di Bologna, 40138 Bologna, Italy; 4 Institute of Genetics and Biomedical Research – Milan Unit, National Research Council of Italy, 20089 Rozzano, (MI) Italy; 5 Department of Morphology, Surgery and Experimental Medicine, University of Ferrara, 44121 Ferrara, Italy; 6 Department of Experimental, Diagnostic and Specialty Medicine (DIMES), University of Bologna, 40138 Bologna, Italy; 7 Nephrology, Dialysis and Renal Transplant Unit, St. Orsola Hospital, IRCCS Azienda Ospedaliero-Universitaria di Bologna, 40138 Bologna, Italy; 8 Department of Medico-Surgical Sciences and Biotechnologies, University of Rome Sapienza, 04100 Latina, Italy; 9 Mediterranea Cardiocentro, 80122 Naples, Italy; 10 Department of Public Health – Nephrology Unit, University of Naples Federico II, 80131 Naples, Italy; 11 Department of Advanced Biomedical Sciences, University of Naples Federico II, 80131 Naples, Italy

**Keywords:** Prognostic markers, Cardiovascular diseases

## Abstract

Enzyme replacement therapy (ERT) is a mainstay of treatment for Anderson–Fabry disease (AFD), a pathology with negative effects on the heart and kidneys. However, no reliable biomarkers are available to monitor its efficacy. Therefore, we tested a panel of four microRNAs linked with cardiac and renal damage in order to identify a novel biomarker associated with AFD and modulated by ERT. To this end, 60 patients with a definite diagnosis of AFD and on chronic ERT, and 29 age- and sex-matched healthy individuals, were enrolled by two Italian university hospitals. Only miR-184 met both conditions: its level discriminated untreated AFD patients from healthy individuals (c-statistic = 0.7522), and it was upregulated upon ERT (*P* < 0.001). On multivariable analysis, miR-184 was independently and inversely associated with a higher risk of cardiac damage (odds ratio = 0.86; 95% confidence interval [CI] = 0.76–0.98; *P* = 0.026). Adding miR-184 to a comprehensive clinical model improved the prediction of cardiac damage in terms of global model fit, calibration, discrimination, and classification accuracy (continuous net reclassification improvement = 0.917, *P* < 0.001; integrated discrimination improvement [IDI] = 0.105, *P* = 0.017; relative IDI = 0.221, 95% CI = 0.002–0.356). Thus, miR-184 is a circulating biomarker of AFD that changes after ERT. Assessment of its level in plasma could be clinically valuable in improving the prediction of cardiac damage in AFD patients.

## Introduction

Anderson–Fabry disease (AFD) is a rare X-linked lysosomal storage disorder caused by mutations of the alpha-galactosidase A gene (*GLA*), located on the X chromosome (Xq22.1). Deficiency of GLA causes accumulation of a neutral glycosphingolipid, globotriaosylceramide (Gb3), in the lysosomes of various tissues and organs, including the vascular endothelium, kidneys, heart, eyes, skin, and nervous system [1]. The spectrum of clinical presentation is extremely broad. The disease has an early onset, usually in childhood, and its classical phenotype is characterized by neuropathic pain, angiokeratomas, cornea verticillata, and gastrointestinal disturbances [2]; after the third decade of life, cardiac involvement, renal failure, and cerebrovascular events may occur and are the major causes of morbidity and mortality [3]. In contrast, non-classical AFD presents with a milder, later onset and a variable phenotype, usually with the manifestation of cardiac disease. In addition, as a consequence of random X-chromosome inactivation (lyonization), heterozygous females present with variable clinical manifestations, ranging from an almost absence of symptoms to very severe pathologies similar to those observed in males, albeit usually with a later onset [4].

Enzyme replacement therapy (ERT) and oral pharmacologic chaperones are the specific treatments for AFD. The most-used recombinant enzyme (either agalsidase alpha or beta) is intravenously administered to restore missing enzymatic function, so reducing the accumulation of Gb3 in tissues and slowing down disease progression [5]. Pharmacologic intervention before the occurrence of irreversible manifestations is crucial for improving biochemical response and outcome [6]. Indeed, organ transplant may represent the only therapeutic option for advanced AFD.

Recently, globotriaosylsphingosine (lyso-Gb3), which accumulates in the plasma of AFD patients, has been proposed as a diagnostic biomarker [7] and has become part of a panel of variables used to decide the optimal time to initiate therapy [8–10]. However, the relationship between lyso-Gb3 and organ damage is still uncertain [11]. The discovery of circulating biomarkers associated with organ damage and modulated by ERT could be clinically useful in improving risk stratification and monitoring ERT efficacy in AFD patients. Small non-coding RNAs, such as microRNAs (miRNAs), could be used for this purpose. They are acknowledged to regulate gene expression and have been implicated in many physiological and pathological processes, like cell regulation [12], cancer [13], cardiovascular diseases [14], and renal dysfunction [15]. Several miRNAs have been shown to contribute to the development and progression of cardiovascular diseases, including heart failure [16], myocardial infarction [17], cardiomyopathies [18], arrhythmias [19], and atherosclerosis [20], as well as renal conditions [21]. Moreover, miRNAs are secreted as part of the cargo of microvesicles or exosomes from a variety of cell types, are released from damaged cells, and can be taken up by recipient cells [22]. Importantly, when they are released into body fluids, they are protected from RNase-mediated degradation by multiple mechanisms (e.g., formation of miRNA–protein complexes), leading them to be stable and, hence, detectable [23]. For this reason, they have been proposed as biomarkers of cardiovascular pathology [24], including those associated with AFD [25, 26].

Here, we selected a panel of miRNAs potentially associated with cardiac and renal damage as candidate biomarkers of AFD and ERT response. We first assessed if the circulating levels of these miRNAs were associated with AFD and modulated by ERT. Then, we evaluated the independent association of any promising miRNAs with organ damage and any additional predictive value when added to a clinical model of AFD.

## Materials and methods

### Setting

Sixty patients (35 males; 25 females) with a definitive diagnosis of AFD [27] comprised the whole study cohort: 37 were enrolled by Azienda Ospedaliero-Universitaria Policlinico Federico II, Naples, and 23 by the Cardiology Unit, IRCCS Azienda Ospedaliero-Universitaria di Bologna, Bologna, Italy. Except for those enrolled at first diagnosis (*N* = 16), all patients were already on chronic ERT. At the time of enrollment, all subjects underwent a complete clinical examination. Blood samples were systematically collected and analyzed for high-sensitivity troponin I (TnI) and pro-B-type natriuretic peptide (pro-BNP) levels [28]. Plasma lyso-Gb3 levels were measured by liquid chromatography-mass spectroscopy. The study protocol conformed to the ethical guidelines of the 1975 Declaration of Helsinki and was approved by the review board of the institutions involved (protocol #ICH-967); written informed consent was obtained from all enrolled individuals.

### Blood collection for RNA purification

Five ml of peripheral blood was collected in EDTA-containing Vacutainer tubes for plasma separation. Total RNA, including miRNA, was extracted from plasma with the miRNeasy Mini Kit (cat. no. 217004, Qiagen), as described elsewhere [29], and used for droplet digital PCR (ddPCR) testing.

### Reverse transcription and droplet digital PCR

Reverse transcription of 8 µl of RNA was performed using the miRCURY LNA RT Kit (Qiagen, cat. no. 339340), following the manufacturer’s protocol. Absolute levels of circulating miR-184 were quantified using ddPCR (Bio-Rad). The QX200 AutoDG Droplet Digital PCR System was used for the amplification and detection of the target miRNA, following a protocol we developed [30]. An EvaGreen-based assay, in association with an LNA primer (Exiqon-Qiagen), was chosen to test miRNA expression. We used the following miRCURY LNA PCR primer set (Exiqon-Qiagen): hsa-miR-1–3p (YP00204344); hsa-miR-133a-3p (YP00204788), hsa-miR-146a-5p (YP00204688), hsa-miR-184 (YP00204601). PCR was performed using constant RT volumes in a 20 µl reaction volume.

### Study endpoint and definitions

The study’s primary endpoint was cardiac damage, defined as increased LV mass. LV mass was normalized for the height-to-allometric signals (LVMh) parameter: a cut-off of 50 g/m^2.7^ for males (M) and 47 g/m^2.7^ for females (F) was used to discriminate patients with or without cardiac damage [31, 32]. Other echocardiographic parameters were defined as follows: left ventricular hypertrophy (LVH) upon the presence of a maximum LV wall thickness ≥ 13 mm in end-diastole in the absence of any other systemic or cardiac process capable of producing LVH [33], on standard trans-thoracic echocardiography; maximal wall thickness (MWT) was defined as the greatest ventricular thickness, obtained at end-diastole and in short-axis views, measured at any site in the LV wall; LV mass indexed for body surface area (LVMi) was calculated by the Devereux formula on the basis of echocardiographic measurements; ejection fraction (EF) was calculated using the modified Simpson method; blood pool pulsed Doppler of mitral valve inflow was used to extract the ratio of early-to-late diastolic flow velocity and the deceleration time [34]; left atrial volume indexed for body surface area (LAVi) was measured by Simpson’s biplane method, with a cut-off of 34 ml/m^2^ considered to indicate left atrial enlargement [35]; and LV longitudinal function was assessed by two-dimensional speckle tracking measurements of LV global longitudinal strain (GLS) taken in the 2-, 3-, and 4-chamber apical views [36]. The endocardial border was traced at end-systole and manually adjusted to include the entire myocardial wall.

Renal function was evaluated via estimated glomerular filtration rate (eGFR) and amount of protein excretion in urine: eGFR was calculated using the CKD-EPI formula for adults and the Schwartz formula for children up to 18 years of age; albuminuria and proteinuria excretion were categorized following Kidney Disease Improving Global Outcomes guidelines [37–39].

Classical AFD phenotype was defined based on *GLA* mutation type, organ or multi-organ involvement, and time of disease onset.

### Statistical analyses

Categorical data are expressed as proportions, and continuous variables are reported as medians and interquartile ranges (25th–75th percentiles). For comparisons between groups, the Mann–Whitney test was used for continuous variables and the Chi-square test for categorical variables. In the validation cohort, the area under the receiver operating characteristic (ROC) curves (c-statistic) was used to evaluate the diagnostic performance of candidate miRNAs in differentiating AFD from control patients. Non-parametric Wilcoxon matched-paired signed-rank test was used to compare continuous variables before and after ERT. For the whole study cohort, we also used Spearman’s rank correlation coefficient to evaluate the correlation between a selected miRNA and clinical variables.

The relation between a selected miRNA and cardiac damage was investigated with the use of a backward multivariable logistic regression model adjusted for variables selected at univariable analysis (*P* < 0.1). The additional contribution of the selected miRNA to the model for the prediction of study end-point was evaluated in terms of global model fit, calibration, discrimination, and classification accuracy. The likelihood-ratio test was used for global model fit. Model discrimination was assessed using the c-statistic. Calibration was evaluated by the Hosmer Lemeshow test, with a χ^2^ ≥ 20 (*P* < 0.01) indicating poor calibration. Classification accuracy was evaluated by measuring continuous net reclassification improvement (NRI) > 0, integrated discrimination improvement (IDI), and relative IDI (rIDI) [40]. For the last index, 95% confidence intervals (CIs) were calculated using bootstraps estimation. Continuous NRI defined any change in predicted probability as either upward or downward movement, depending on the direction, whereas IDI took into account a weight for each movement and was equal to the difference in discrimination slopes [41]. The rIDI indicated the increase in discrimination slopes divided by the slope of the model without the selected miRNA. A *P* < 0.05 in the two-tailed tests was considered significant. All analyses were performed with STATA 14.0 software (STATA Corporation).

## Results

### Identification of candidate circulating miRNA biomarkers

Four miRNAs were selected as potential biomarkers for AFD due to their role in cardiac and renal damage: hsa-miR-1–3p [42, 43], hsa-miR-133a-3p [44, 45], hsa-miR-146a-5p [46], and hsa-miR-184 [47, 48]. A pilot screening conducted on a group of patients sampled before and after ERT administration (*N* = 12) led to the selection of miR-184 as the most promising biomarker of response to ERT (Supplementary Fig. S1). Quantification of the miRNAs in all patients revealed that miR-184 and miR-146a-5p were differentially expressed in AFD patients *vs*. control individuals (respectively, *N* = 60 and *N* = 42) (Supplementary Fig. S2). Accordingly, miR-184 was selected as the candidate biomarker for further analyses, being both associated with AFD and modulated by ERT.

### Validation of miR-184 as an AFD biomarker

**Fig. 1:**
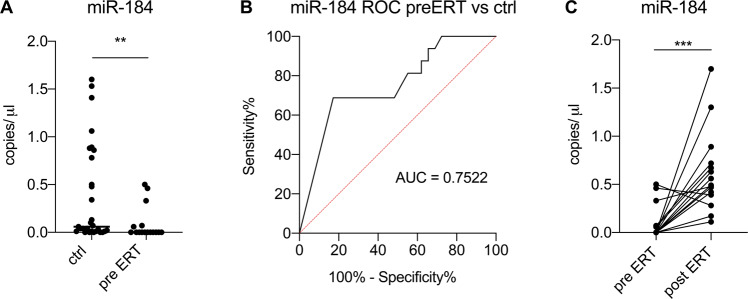
miR-184 as a biomarker in AFD. **A** Circulating levels of miR-184, measured by ddPCR, in AFD patients before starting treatment (pre-ERT) and in healthy controls (ctrl) (*N* = 16 pre-ERT patients; *N* = 29 healthy controls). **P < 0.05 (non-parametric Mann–Whitney test). **B** ROC curve and relative area under the curve calculation for miR-184 as a biomarker of AFD. **C** miR-184 is modulated by ERT. The circulating level of miR-184, measured by ddPCR, in 16 AFD patients before (pre-ERT) and after (post-ERT) the start of ERT. ***P = 0.0004 (non-parametric Wilcoxon matched-paired signed-rank test).

Circulating levels of miR-184 were quantified by ddPCR in 16 AFD patients before and after ERT and in 29 healthy individuals. ROC curve analysis and relative AUC were used to evaluate the diagnostic performance of miR-184 in discriminating AFD patients from healthy controls (Fig. 1A, B). Paired analysis of pre- *vs*. post-ERT clinical characteristics and laboratory variables, including miR-184 level, revealed that the miRNA was significantly increased upon therapy and that there were no significant changes in any other clinical variable except for lyso-Gb3, which was modulated to a minor extent (Table 1 and Fig. 1C). Therefore, miR-184 was confirmed to be associated with AFD and modulated by ERT.

**Table 1:** Clinical characteristics of 16 AFD patients before and after ERT.

Variable	pre-ERT^a^	post-ERT^a^	*P* Value
HR, bpm	73 [64–85]	74 [66–82]	0.349
SBP, mmHg	120 [110–130]	120 [110–125]	0.287
DBP, mmHg	75 [70–80]	75 [70–80]	0.903
BMI, kg/m^2^	24.2 [22.2–26.47]	24.2 [22.7–27.4]	0.059
miR-184, copies/μl	0 [0–0.065]	0.525 [0.404–0.805]	0.001
proBNP, pg/ml	34.1 [9.1–234.1]	23.84 [14.12–104.3]	0.570
eGFR, ml/min	120.5 [107–125]	120 [107–129]	0.195
lysoGb3, ng/ml	3.1 [1.2–9.3]	2.2 [0.7–3.5]	0.010
tdIVS, mm	10 [8.5–12]	11 [9–13]	0.156
tdPW, mm	9 [8–12]	9 [8–13]	0.739
EDV, ml	120 [81–143]	120 [93–134]	0.594
ESV, ml	43 [30–51]	41 [37–50]	0.533
EF, %	0.64 [0.59–0.69]	0.62 [0.55–0.65]	0.539
LVMi, g/m^2^	90 [77–118]	91.2 [86.1–106]	0.753
LVMh, g/m^2.7^	39.0 [32.0–51.5]	39.1 [36.8–50.3]	0.799
LAVi, ml/m^2^	28 [22.98–31]	30 [28–33]	0.074
sPAP, mmHg	26 [25–35]	26 [25–28]	0.144
GLS, %	−17.61 [−19.76–−14.95]	−15.8 [−19.04–−11.62]	0.433
TnI, ng/l	0.007 [0.004–0.07]	0.03 [0.003–1.53]	0.230
months of ERT	0	14 [9.5–22]	–

Abbreviations: *MI* Body mass index, *bpm* Beats per minute, *DBP* Diastolic blood pressure, *EDV* End-diastolic volume, *EF* Ejection fraction, *eGFR* Estimated glomerular filtration rate, *ESV* End-systolic volume, *GLS* Global longitudinal strain, *HR* Heart rate, *LAVi* Left atrial volume index; LVEDDi, indexed left ventricular end-diastolic diameter; LVMi/h, left ventricular mass indexed for body surface area/height^2.7^; RWT, relative wall thickness, *SBP* Systolic blood pressure, *sPAP* Systolic pulmonary arterial pressure, *tdIVS* Tele-diastolic interventricular septum, *tdPW* Tele-diastolic posterior wall, *TnI* Troponin. ^a^Data are median [interquartile range].

### Prognostic significance of miR-184 and its additional value in predicting cardiac damage in AFD

Spearman’s correlation analyses revealed that miR-184 negatively correlated with myocardial mass (LVMi: *r* = −0.31, *P* = 0.02; LVMh: *r* = −0.30, *P* = 0.02), LAVi (*r* = −0.24, *P* = 0.065) and TnI (*r* = −0.6, *P* < 0.01), and positively correlated with kidney function (eGFR: *r* = 0.29, *P* = 0.04); no correlation was observed between miR-184 and lyso-Gb3 (Supplementary Fig. S3).

In our cohort, only 11 patients had clinically relevant renal impairment (eGFR ≤ 60 ml/min); in contrast, the presence of cardiac damage was around 60%. Patients with cardiac damage were older, had a lower level of miR-184, and were on ERT longer compared to patients without cardiac damage (Table 2). As expected, cardiac mass and function parameters were consistently worse in the former. There was no difference in lyso-Gb3 level between these two sub-groups.

**Table 2:** Clinical characteristics of 60 AFD patients under chronic ERT stratified for the presence of cardiac damage.

Variable	All population^a^ (*N* = 60)	Cardiac damage^a^ (*N* = 33)	No cardiac damage^a^ (*N* = 27)	*P* Value
Age, years	45 [32–55]	54 [42–62]	35 [23–47]	**<0.001**
Males, *n* (%)	35 (58.3)	22 (66.7)	13 (48.1)	0.191
Months on ERT	23 [14–60]	36 [22–68]	15 [10–24]	**<0.001**
HR, bpm	70 [63–80]	67 [60–80]	70 [65–80]	0.203
SBP, mmHg	120 [110–130]	120 [110–130]	120 [115–130]	0.798
DBP, mmHg	80 [70–80]	80 [70–80]	80 [70–80]	0.607
BMI, kg/m^2^	25.5 [23.0–28.4]	26.8 [23.9–29.1]	23.9 [22.3–26.7]	0.022
BSA, m^2^	1.8 [1.7–2.0]	1.85 [1.73–1.93]	1.8 [1.6–2.0]	0.275
miR-184, copies/μl	0.40 [0.01–4.50]	0.03 [0.00–3.0]	0.63 [0.11–10.5]	**0.014**
proBNP, pg/ml	103.7 [76.8–355.1]	258.3 [101.0–1713.4]	84 [23.84–104.30]	**<0.001**
eGFR, ml/min	90.5 [72.8–111.0]	81.0 [51.8–91.0]	110 [98.5–123.0]	**<0.001**
lysoGb3, ng/ml	2.6 [1.3–8.1]	4.55 [1.25–10.85]	2.20 [1.30–3.70]	0.140
tdIVS, mm	12 [10–15]	15 [13–17]	10 [9–11]	**<0.001**
tdPW, mm	11 [9–13]	13 [12–15]	9.0 [8.8–9.8]	**<0.001**
EDV, ml	98 [79–120]	100 [82–130]	95.0 [73.5–108.0]	0.190
ESV, ml	35 [27–42]	38 [27–47]	31.5 [26.5–40.5]	0.244
EF, %	0.67 [0.63–60]	0.67 [0.63–62.0]	0.66 [0.64–30.36]	0.668
LVMi, g/m^2^	127.6 [91.7–167.0]	161 [134–194]	90.8 [80.9–98.0]	**<0.001**
LVMh, g/m^2.7^	51.2 [40.6–70.3]	66.8 [54.3–88.0]	39 [34.8–44.2]	**<0.001**
LAVi, ml/m^2^	33.9 [28.0–43.0]	41 [37–60]	28.5 [25.1–32.3]	**<0.001**
sPAP, mmHg	29.5 [25.0–36.0]	35 [28–40]	26.0 [25.0–29.5]	**<0.001**
GLS, %	−15.8 [−19.0–−11.8]	−12.7 [−16.6–−8.5]	−18.3 [−20.8–−15.8]	**<0.001**
TnI, ng/l	0.09 [0.01–5.50]	0.20 [0.07–26.0]	0.01 [0.00–0.83]	**<0.001**

Abbreviations: As in Table 1.

^a^Data are median [interquartile range], unless stated otherwise.

Multivariable logistic analyses revealed an independent and inverse relationship between miR-184 and the risk of cardiac damage (Table 3). Of note, although not associated with the study endpoint at the bivariate level, we forced lyso-Gb3 level into the multivariable model. The variable was not retained by the backward stepwise selection method, and the main study findings did not change.

**Table 3:** Independent predictors of cardiac damage in 60 AFD patients on ERT (multivariable logistic regression).

	Odds ratio	95% Conf. interval	*P* Value
**miR-184**	0.864	0.7598–0.983	0.026
**Classical phenotype**	10.691	1.379–82.908	0.023
**Months of ERT**	1.017	0.997–1.038	0.089
**eGFR**	0.922	0.877–0.970	0.002

Finally, we estimated the contribution of miR-184 to a clinical model for the prediction of cardiac impairment. The addition of miR-184 to other variables independently associated with cardiac damage (disease phenotype, eGFR, and ERT duration) improved the model’s performance in terms of global fit, discrimination power (although not to a statistically significant extent), and classification accuracy (Table 4). In this regard, classification accuracy improved by 66% in patients with cardiac damage and by 25% in patients without damage, leading to a continuous NRI ( > 0) of 91.7%. Although improvement in classification accuracy was mitigated by indexes that took into account the weight of any change in predicted probabilities, such as IDI and rIDI, it remained statistically significant.

**Table 4:** Additional contribution of miRNA-184 to a clinical model for the prediction of cardiac damage.

	Model without miR-184	Model with miR-184
Global model fit:		
Likelihood ratio chi-square test	30.31	37.56
Likelihood ratio test	*P* = 0.007	
Calibration:		
Hosmer–Lemeshow, chi-square	14.48 *P* = 0.070	10.78 *P* = 0.214
Discrimination:		
c-statistic	0.889	0.925
Δ c-statistic	0.036 (*P* = 0.250)	
Classification accuracy:		
Continuous NRI ( > 0)	0.917 (*P* < 0.001)	
IDI	0.105 (*P* = 0.017)	
Relative IDI (95% bootstrap C.I.)	0.221 (0.002–0.356)	

Global model fit, calibration, discrimination, and classification accuracy of logistic regression models.

Abbreviations: *IDI* Integrated discrimination improvement, *NRI* Net reclassification improvement.

## Discussion

In this study, we determine whether miR-184 could serve as a biomarker for risk stratification and the monitoring of treatment efficacy. The main findings of the present study were as follows: (1) the level of miR-184 is associated with AFD and is also modulated by ERT; (2) a higher circulating level of miR-184 is independently associated with a lower risk of cardiac damage in AFD patients; and (3) adding miR-184 to a comprehensive clinical model improves the prediction of cardiac damage in AFD patients. These results suggest that assessment of the plasma level of miR-184 could be of use for assessing response to ERT and the prognosis of AFD.

AFD manifests with a range of severities and a heterogeneous spectrum of phenotypes: whereas hemizygous male patients typically present with severe symptoms, the clinical manifestations of heterozygous female patients depend on the *GLA* variant and the lyonization pattern in tissues [49]. Premature death occurs mostly due to cardiac complications and end-stage renal disease. ERT—which is based on intravenous infusions of recombinant forms of alpha-galactosidase—is an expensive and lifelong treatment option that has been available since 2001 [50]. The timing of treatment initiation is critical: indeed, although ERT can slow disease progression, it cannot reverse permanent damage, at which point organ transplant is the only therapeutic option. Thus, initiating therapy prior to irreversible cellular impairment is crucial for mitigating damage caused by Gb3 deposition [51]. As a matter of fact, supplementing GLA activity in order to improve Gb3 clearance was not sufficient to rescue profibrotic signaling and dysregulated autophagy in a podocyte culture model of AFD [52, 53]. The use of lyso-Gb3 as a biomarker of AFD and for monitoring of ERT outcome needs to be better investigated since additional mechanisms besides Gb3 accumulation may be taking place in AFD [54]. Recently, elevated plasma levels of several proteins, including inflammatory and cardiac remodeling biomarkers, have been reported in AFD patients [55]. Among circulating nucleic acids, some miRNAs were proposed as circulating biomarkers for AFD diagnosis and prognosis, either in plasma [26] or serum [25], but the number of samples in the studies was low, and no coupled analysis of pre- and post-ERT patients were reported.

Here, we conducted a discovery study on a panel of four candidate miRNAs. In our cohort of 60 patients enrolled at two different hospitals, we found miR-184 to be significantly reduced in untreated AFD patients *vs*. healthy individuals. Moreover, this miRNA was modulated by ERT when other clinical variables were still mostly unaltered shortly after initiation of ERT. Therefore, miR-184 is a sensitive circulating biomarker that is modulated in response to ERT administration. Furthermore, we found that a lower miR-184 level is independently associated with the risk of cardiac damage, and that the incorporation of miR-184 into a clinical model improves risk prediction of impaired cardiac function, as assessed by statistical metrics. These results indicate that miR-184 is a circulating biomarker that could be employed in the clinic for assessing the response to ERT as well as the severity of the disease.

MiR-184 has been reported in several studies to have a role in response to the stress of cardiac and renal tissue. The miRNA was found downregulated in cardiac hypertrophy. Indeed, through competitive binding with the long noncoding RNA *UCA1*, miR-184 controls the mRNA level of homeobox A9, inducing upregulation of atrial and brain natriuretic peptide genes in hypertrophic cardiomyocytes [56]. Exposure to radical oxygen species was found to induce oxidation-dependent modification of the miR-184 level to regulate apoptosis through the downregulation of Bcl-xL and Bcl-w in a mouse model of myocardial ischemia–reperfusion [47]. The miRNA was also found expressed in renal tubules, in which it induced fibrosis through downregulation of the phospholipid phosphatase three gene, and its expression was found to be controlled by albumin, triggering the recruitment of NF-kB to the miR-184 promoter in diabetic nephropathy [48]. Importantly, miR-184 is released into the circulation, so it can serve as a disease biomarker [57].

In our study cohort, we did not observe any relationship between the circulating level of lyso-Gb3 and the risk of cardiac damage. The reason for this finding is unclear, but it may be related to our small sample size. Indeed, lyso-Gb3 is a powerful diagnostic biomarker and has been shown to correlate with phenotype and organ damage severity in patients with AFD [58, 59]. However, a relation between lyso-Gb3 changes over time and treatment outcome (such as in LV mass or eGFR) has not been validated until now. Neither lyso-Gb3 concentration at baseline or during treatment nor its absolute decrease have been shown to predict clinical events or changes in organ damage [2]. Additionally, the baseline lyso-Gb3 has not emerged as a predictor of myocardial fibrosis during follow-up in untreated patients [60]. Therefore, prognostic biomarkers for AFD progression and clinical response to guide treatment decisions are not available at the moment.

AFD induces multi-organ failure via progressive deposition of unprocessed material inside the lysosome, which in the heart leads to hypertrophy and activation of other intracellular processes associated with inflammation, apoptosis, and pro-oxidative molecule production [61]. Given that our patient cohort presented mainly with the classical form of the pathology and showed predominantly cardiac involvement, with only mild kidney damage, we put forward that the circulating level of miR-184 is at least a sensitive and early sensor of cardiac impairment, and therefore that its measurement could be clinically relevant for discriminating patients with cardiac damage due to chronic intracellular glycosphingolipid accumulation.

### Limitations

Owing to our small sample size, we could have missed some other significant relationships, such as that between lyso-Gb3 and cardiac damage (as mentioned above). However, to the best of our knowledge, the present study comprises more patients than previous ones and introduces the novelty of measuring a panel of miRNAs pre- and post-ERT. Moreover, AFD is associated with the risk of cardiac and renal impairment. Although we found that miR-184 level positively correlated with eGFR, the number of patients with clinically relevant renal dysfunction was very low (*N* = 11). This prevented us from building a multivariable model for renal damage prediction because of the risk of overfitting.

Finally, our observation period of post-ERT samples was limited to one time-point, so the complete picture of disease progression is still needed. Future investigations will include multiple samplings throughout the course of the disease, a dose–response analysis of ERT, and a longer clinical follow-up to establish the relationship between changes in circulating miR-184 level and disease progression. Furthermore, more studies are required to elucidate whether this miRNA targets specific molecular pathways inside cells, playing a role in AFD pathogenesis.

### Perspectives

Monitoring the efficacy of ERT is still problematic in the management of AFD. Indeed, we know of no circulating biomarker that can strongly indicate whether and how ERT is working, anticipating the mid- to long-term clinical outcomes of the therapy. In the present study, we have identified a circulating microRNA, namely miR-184, as a sensitive biomarker of ERT that adds value to clinical and biochemical parameters in discriminating significant cardiac involvement in disease progression. Whether changes in the miR-184 level associated with ERT could translate into better clinical outcome should be investigated in properly powerful studies.

## Supplementary information

Supplemental file

AU CONTRIBUTION FORM

## Data Availability

The droplet digital PCR data analyzed in the current study are available from the corresponding authors on reasonable request.
