# Supplementary material for: Circulating miR-184 is a potential predictive biomarker of cardiac damage in Anderson–Fabry disease
Source: Cell Death Dis. 2021 Dec 11;12(12):1150. doi: 10.1038/s41419-021-04438-5 (PMC8665928; doi:10.1038/s41419-021-04438-5)
Supplement: Supplementary file 1 — Supplemental file [file 41419_2021_4438_MOESM1_ESM.pdf]

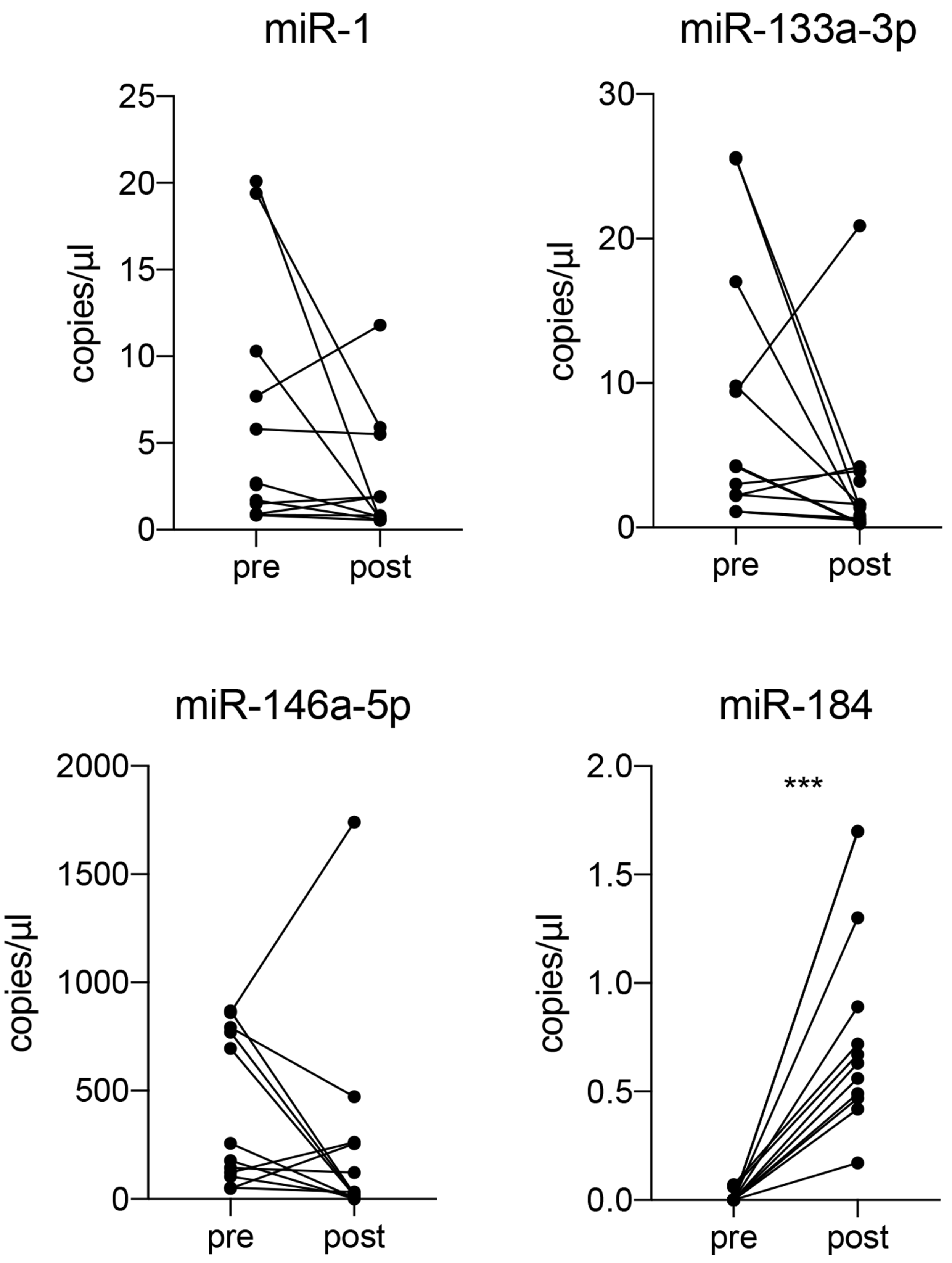

Fig. S1. Panel of miRNAs tested at a first screening. Quantification of four microRNAs through ddPCR in 12 patients before (pre) and after (post) the start of ERT. *, P<0.5; **, P<0.01; ***, P < 0.001 (non-parametric Wilcoxon matched-paired signed rank test).

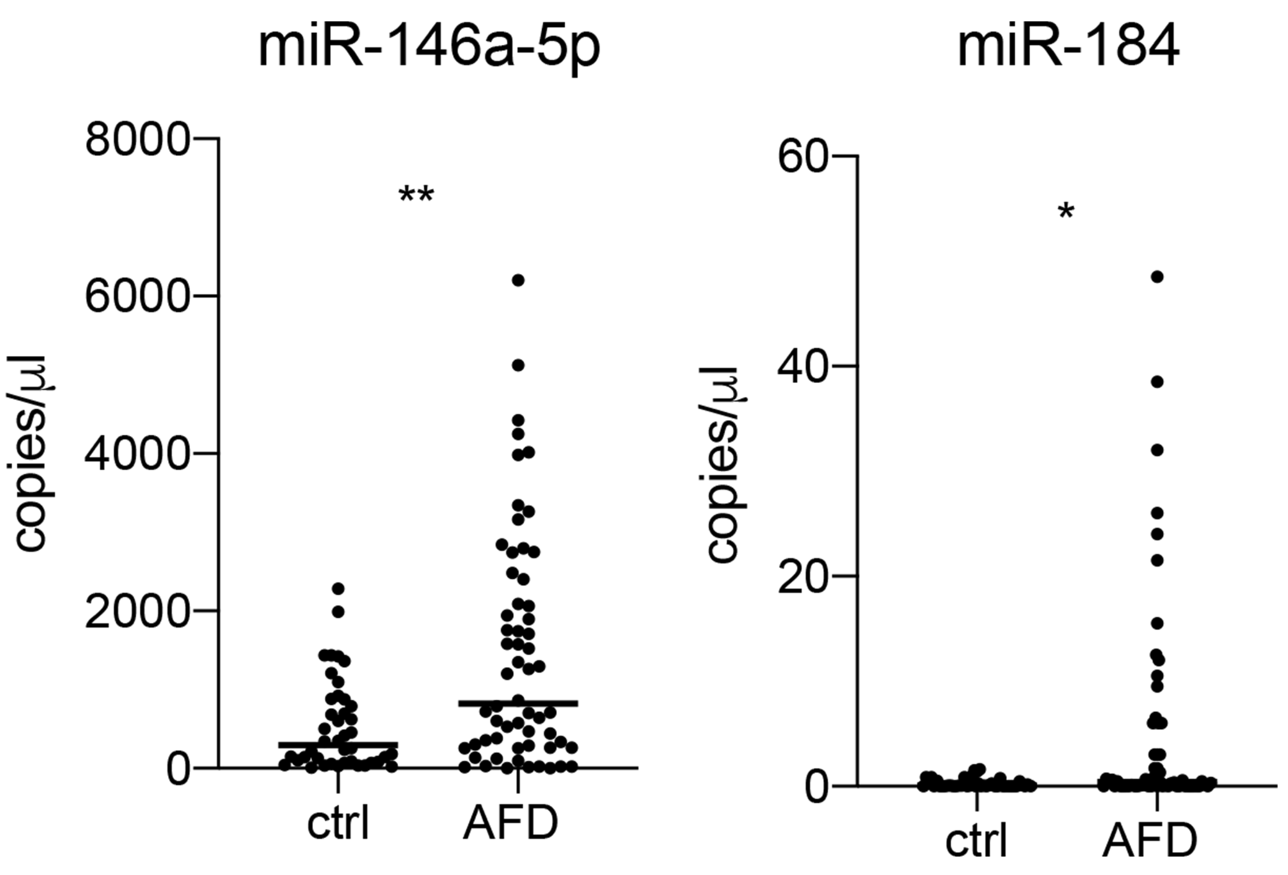

Fig. S2. Panel of tested miRNAs in all patients and controls. Quantification of two selected miRNAs throu-gh ddPCR in 60 AFD patients and 42 control individuals. *, P<0.5; **, P<0.01; ***, P < 0.001 (non-parametric Mann Whitney test).

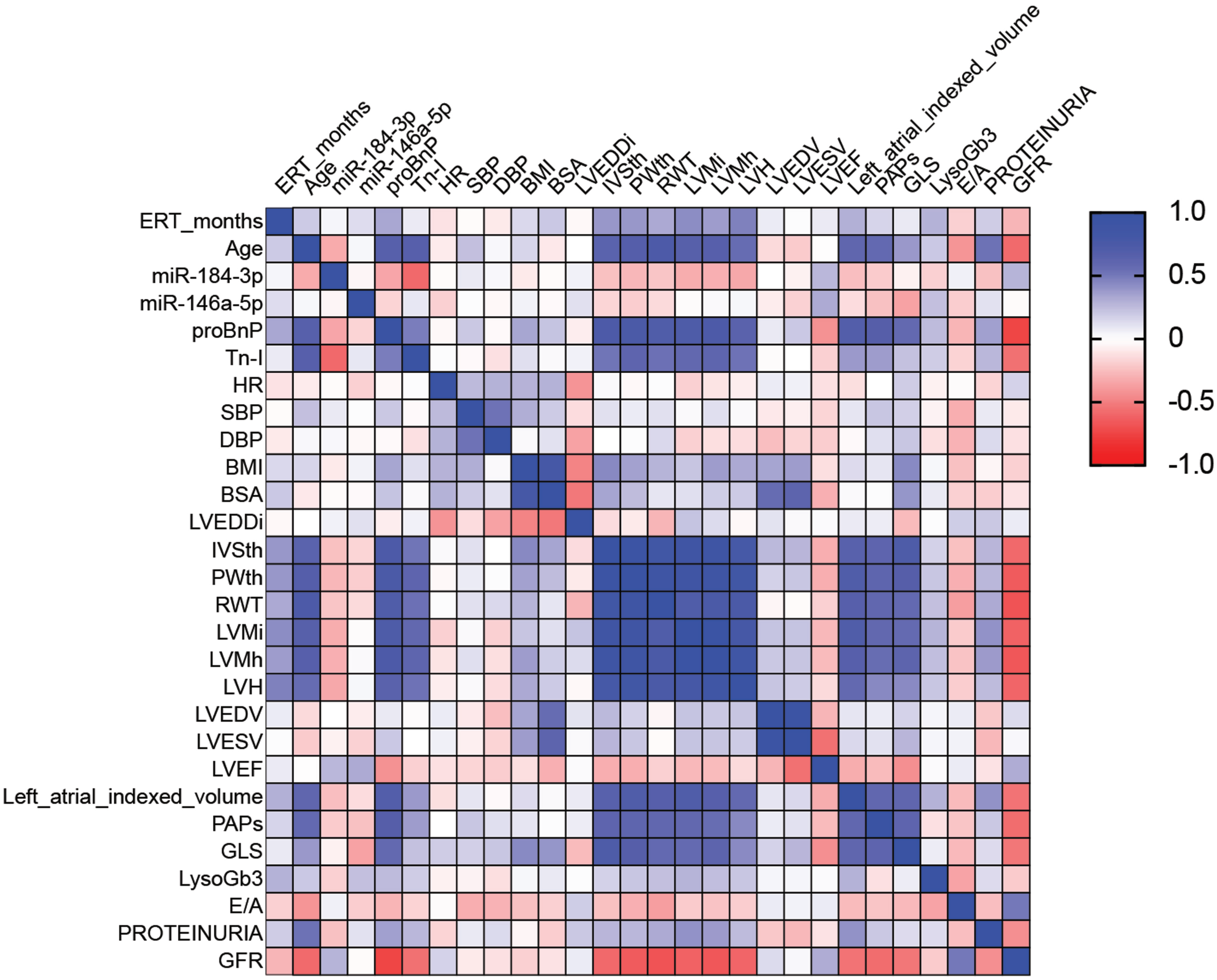

Fig. S3. Heatmap of Spearman's correlation analyses. Heatmap of correlations between miR-184 and different clinical parameters evaluated in this study.
