# Supplementary material for: Circulating miR-184 is a potential predictive biomarker of cardiac damage in Anderson–Fabry disease
Source: Cell Death Dis. 2021 Dec 11;12(12):1150. doi: 10.1038/s41419-021-04438-5 (PMC8665928; doi:10.1038/s41419-021-04438-5)
Supplement: Supplementary file 2 — AU CONTRIBUTION FORM [file 41419_2021_4438_MOESM2_ESM.pdf]

DECLARATION OF CONTRIBUTIONS TO ARTICLE

ADMC

Manuscript Number: CCDIS-21-2570

Journal Name: *Cell Death & Disease* (the 'Journal')

Proposed Title of the Contribution:

Circulating miR-184 is a Potential Predictive Biomarker of Cardiac Damage in Anderson-Fabry Disease (the 'Contribution)

Author(s):

Irene Salamon,Elena Biagini, Paolo Kunderfranco, Roberta Roncarati, Manuela Ferracin, Nevio Taglieri, Elena Nardi, Noemi Laprovitera, Luciana Tomasi, Marisa Santostefano, Raffaello Ditaranto, Giovanni Vitale, Elena Cavarretta, Antonio Pisani, Eleonora Riccio, Valeria Aiello, Irene Capelli, Gaetano La Manna, Nazzareno Galiè, Letizia Spinelli, Gianluigi Condorelli (the 'Authors')

For all *CDDis* articles, each person named as an author in the published version must be able to show he or she has contributed substantially to the article.

Authorship credit should be based on 1) substantial contributions to conception and design, acquisition of data, or analysis and interpretation of data; 2) drafting the article or revising it critically for important intellectual content; and 3) final approval of the version to be published. Authors should meet conditions 1, 2 and 3.

Any person who cannot be shown to have made a substantial contribution to the article cannot be listed as an author in the final version. The name of any person who is deemed to have made a minor contribution can, however, appear in the Acknowledgments section of the article.

Please complete the table below to indicate the contributions of all named authors to the manuscript.

Author Full Name:	Specification of Contribution to the Manuscript:
Irene Salamon	1, 2 and 3.
Elena Biagini	1, 2 and 3.
Paolo Kunderfranco	1, 2 and 3.
Roberta Roncarati	1, 2 and 3.
Manuela Ferracin	1, 2 and 3.
Nevio Taglieri	1, 2 and 3.
Elena Nardi	1, 2 and 3.
Noemi Laprovitera	1, 2 and 3.
Luciana Tomasi	1, 2 and 3.
Marisa Santostefano	1, 2 and 3.
Raffaello Ditaranto	1, 2 and 3.
Giovanni Vitale	1, 2 and 3.
Elena Cavarretta	1, 2 and 3.

DECLARATION OF CONTRIBUTIONS TO ARTICLE

ADMC

Manuscript Number:	Journal Name:	
	Cell Death & Disease	(the 'Journal')

Proposed Title of the Contribution:

	(the 'Contribution)

Author(s):

	(the 'Authors')

For all *CDDis* articles, each person named as an author in the published version must be able to show he or she has contributed substantially to the article.

Authorship credit should be based on 1) substantial contributions to conception and design, acquisition of data, or analysis and interpretation of data; 2) drafting the article or revising it critically for important intellectual content; and 3) final approval of the version to be published. Authors should meet conditions 1, 2 and 3.

Any person who cannot be shown to have made a substantial contribution to the article cannot be listed as an author in the final version. The name of any person who is deemed to have made a minor contribution can, however, appear in the Acknowledgments section of the article.

Please complete the table below to indicate the contributions of all named authors to the manuscript.

Author Full Name:	Specification of Contribution to the Manuscript:
Antonio Pisani	1, 2 and 3.
Eleonora Riccio	1, 2 and 3.
Valeria Aiello	1, 2 and 3.
Irene Capelli	1, 2 and 3.
Gaetano La Manna	1, 2 and 3.
Nazzareno Galiè	1, 2 and 3.
Letizia Spinelli	1, 2 and 3.
Gianluigi Condorelli	1, 2 and 3.

Please complete the table below to indicate the contributions of all named authors to the figures.

Figure 1:

IS, EB, RR, MF, NT, EN, NL, LT, MS, RD, GV, EC, AP, ER, VA, IC, GLM, and NG generated and analysed data; PK analysed data and generated statistics; IS, LS, GC analysed data and devised figures.

Figure 2:

Figure 3:

Figure 4:

Figure 5:

Figure 6:

Signed for and on behalf of the Author(s):	Print Name:	Date:
	Gianluigi Condorelli	01/07/2021
